# CT colonography for longitudinal in-vivo assessment of colonic lengthening in middle-age and older adults

**DOI:** 10.1007/s00261-025-05334-8

**Published:** 2025-12-19

**Authors:** Perry J Pickhardt, Varun Razdan

**Affiliations:** Department of Radiology, University of Wisconsin School of Medicine & Public Health, Madison, USA

**Keywords:** CT colonography, Large intestine, Colon, Anatomy, Virtual colonoscopy

## Abstract

**Background:**

Although a general trend for longer colons among older adults has been observed, longitudinal demonstration of intra-patient elongation is lacking. We assessed colonic length at serial CT colonography (CTC), which allows for precise length measurement over time.

**Methods:**

Longitudinal CTC evaluation (mean time interval, 12.8 years; range, 11.6–16.6 years) in 100 asymptomatic adults (mean age, 54.2 years at index study; 50 men, 50 women) were analyzed using a dedicated software system (V3D Colon, Viatronix). Segmental and total colonic lengths were obtained at interactive 2D and 3D review utilizing the automated luminal centerline.

**Results:**

Mean total colonic length increased from 198.1 cm at index CTC to 205.7 cm at follow-up (*p* < 0.001), corresponding to an annual increase of 0.6 ± 1.2 cm/year. The corresponding median colonic length increased from 191.5 cm to 202.0 cm. Intraperitoneal segments suspended by a mesentery accounted for 80% (6.1 cm) of the average overall 7.6 cm elongation, including a mean increase of 2.1 cm in the sigmoid colon and 4.0 cm in the transverse colon. There was no significant net length change for the extraperitoneal segments (rectum and retroperitoneal colon). Women had a longer colon at initial CTC on average (202.1 cm vs. 194.0 cm) but mean length was similar at final CTC (205.4 cm vs. 206.1 cm) given the increased rate of interval elongation in men (0.88 cm/year in men vs. 0.31 cm/year in women).

**Conclusion:**

Longitudinal CTC showed a mean age-related increase in colonic length of greater than 7 cm per decade in middle-age and older adults. The intraperitoneal colonic segments (transverse > sigmoid) accounted for most of this elongation, whereas the more fixed extraperitoneal segments showed no significant net length increase.

## Introduction

Although a trend for longer colorectal lengths in older adults has been suggested, accurate determination of colonic length is generally difficult with endoscopic, fluoroscopic, laparoscopic, or cadaveric testing [1–4]. In comparison, CT colonography (CTC) represents an ideal modality for assessing colorectal length [5–7]. Using dedicated CTC visualization software, an automated luminal centerline can be derived within the prepared and distended colon, which provides accurate in vivo length measurements, including segmental discrimination. At conventional colonoscopy, telescoping and pleating of the colon artificially foreshorten length measurements [4, 5, 8]. Contrast enema studies are planar displays that can suggest redundancy but cannot account for the complex three-dimensional anatomy of the large intestine for accurate length assessment [2]. Prior cross-sectional CTC studies (i.e., without longitudinal follow-up) have demonstrated that older, thinner, constipated, female patients all tend to have longer colons, on average, as do patients with prior incomplete colonoscopy versus primary CTC screening [5–7, 9].

The purpose of our investigation was to more directly test the hypothesis that colonic length increases over time in adults, using longitudinal intra-patient CTC for precise in-vivo measurement over time, including comparison of intraperitoneal versus extraperitoneal segments.

## Materials and methods

This study was approved by the IRB at the University of Wisconsin-Madison. The requirement for patient consent was waived for this retrospective analysis. The main inclusion criteria consisted of adults who underwent at least two CTC examinations over time, separated by at least a decade between the first and last examinations. In addition, a successful automated luminal centerline to directly measure colonic length was required. To optimize colonic length measurements, the individual series with the best luminal distention among the supine, prone, and decubitus (if performed) positions was chosen [10, 11]. Serial CTC evaluation (mean overall time interval, 12.8 years; range, 11.6–16.6 years) in 100 asymptomatic adults (mean age, 54.2 years at index study; age range, 40–68 years; 50 men, 50 women) meeting these inclusion criteria were analyzed using a dedicated CTC software system (V3D Colon, Viatronix). The first (index) and last available CTC examinations were utilized for length assessment; interim examinations were not included. Sixteen cases where the luminal centerline was unreliable on either the index or follow-up CTC (e.g., due to segmental under-distention) were excluded. A power calculation for study size was not performed as we utilized all available patients meeting the inclusion criteria.

**Fig. 1:**
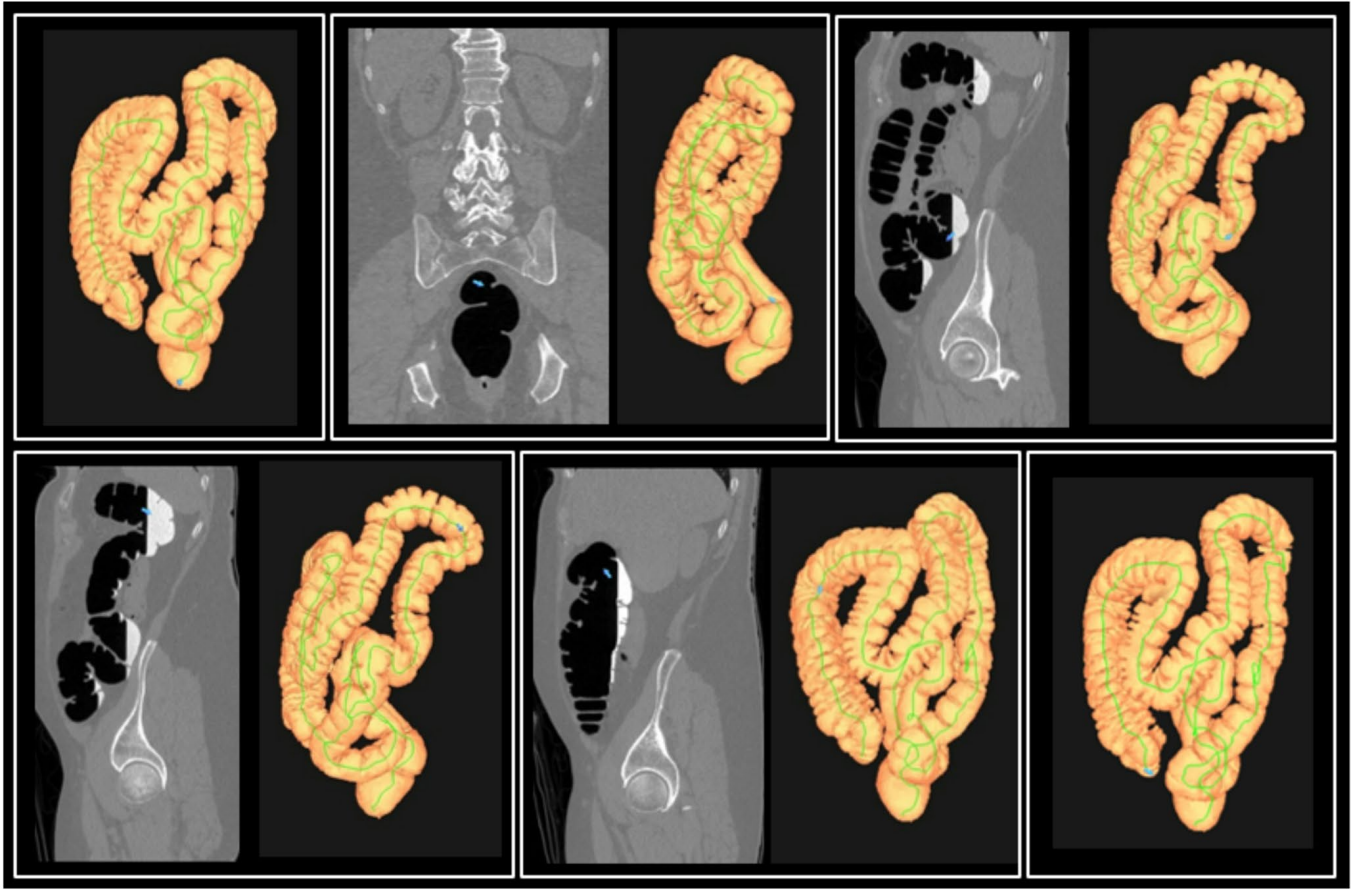
CTC examination in a 69-year-old woman demonstrating the interactive technique for measuring the length of the colorectal segments along the automated centerline.The upper left pane shows the 3D colon map from the supine series with the positional blue arrow located at the anorectal junction. The upper middle pane shows 2D coronal and 3D colon map with blue arrow now at the rectosigmoid junction, as defined by the proximal valve of Houston. The upper right pane shows 2D sagittal and 3D colon map with blue arrow at the junction of the sigmoid and descending colon. The lower left pane shows 2D sagittal and 3D colon map with blue arrow at the splenic flexure, indicating the border between the descending and transverse colon. The lower middle pane shows 2D sagittal and 3D colon map with blue arrow at the hepatic flexure, indicating the border between the transverse and ascending colon. The final 3D map in the lower right pane shows the blue arrow at the cecal tip, which provides the total colonic length (from anorectal junction to cecal tip), which was 191 cm in the case. We did not split up the ascending colon and cecum

In addition to total colorectal lengths, the individual segmental lengths (i.e., rectum, sigmoid colon, descending colon, transverse colon, and ascending colon/cecum) were obtained at interactive 2D and 3D review along the automated centerline, utilizing recognized flexural landmarks (Fig. 1). The ascending colon and cecum were combined for length measurement. The colorectal segments were also grouped into intraperitoneal (transverse and sigmoid colon) and retroperitoneal/extraperitoneal segments for analysis. Total colorectal volumes, which includes both the gas and tagged fluid volumes, were also recorded as a quality assurance step, since degree of distention could conceivably impact length measurements.

Colonic length measurements were initially made by a senior radiology trainee (4 years of experience), and verified by an abdominal radiologist (20 years of experience). The annual interval change in colonic length was calculated as the length difference (in cm) divided by the time interval (in years). Statistical testing for comparing changes in segmental length was performed using the *t* test, as we found that the distribution of colonic length was relatively normal for this cohort. A p-value < 0.05 was considered statistically significant.

## Results

**Fig. 2:**
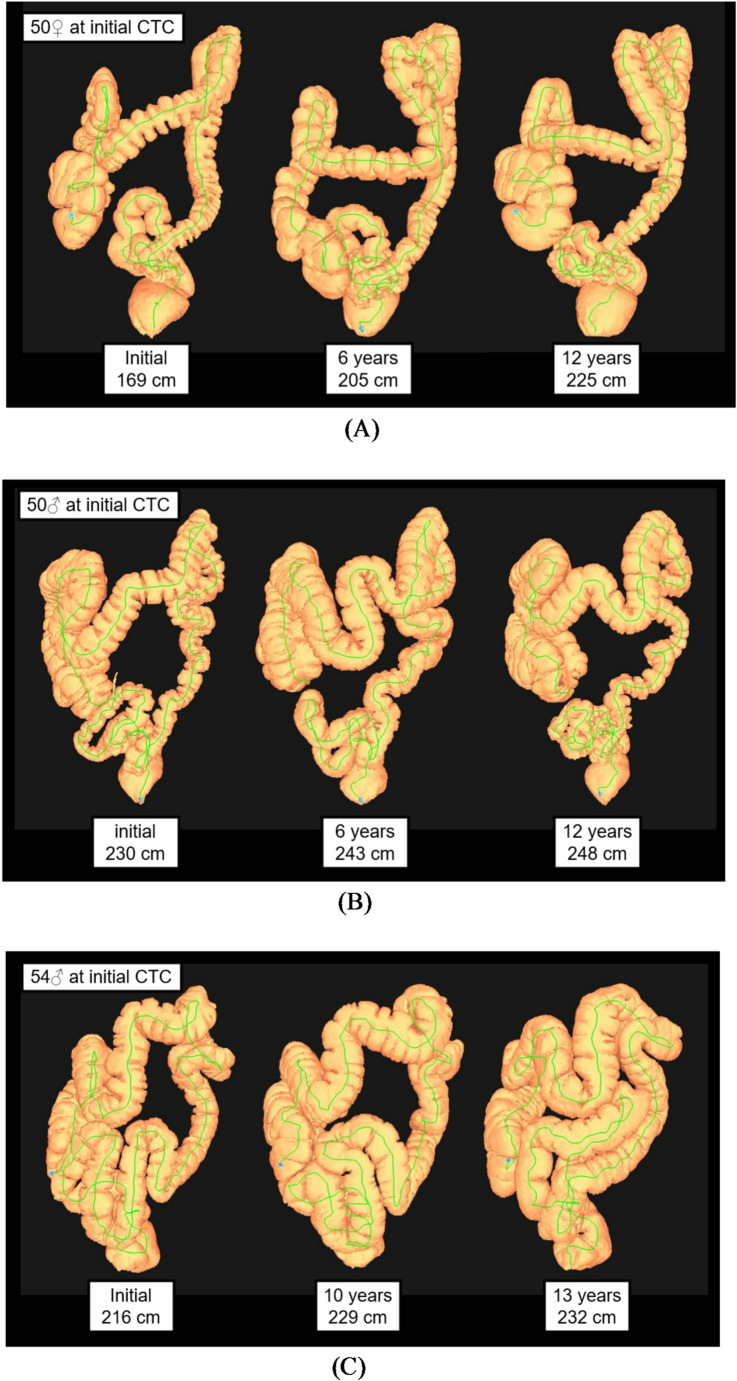
Case examples of colorectal length in patients undergoing longitudinal CTC examinations over time. **A** 3D colon maps from a man who was 50 years of age at initial CTC. Colonic length increased from 169 cm to 225 cm over 12 years. Note how sigmoid and transverse colon contribute the most to the increased in length and redundancy over time. **B** 3D colon maps from a woman who was 50 years of age at initial CTC. Colonic length increased from 230 cm to 248 cm over 12 years. Note again how sigmoid and transverse colon contribute the most to the increased in length and redundancy over time. **C** 3D colon maps from a woman who was 54 years of age at initial CTC. Colonic length increased from 216 cm to 232 cm over 13 years. Note again how sigmoid and transverse colon contribute the most to the increased in length and redundancy over time

The mean total length (± standard deviation) of the large intestine increased from 198.1 cm ± 31.1 cm at index CTC to 205.7 cm ± 34.7 cm at follow-up CTC (*p* < 0.001), for an average increase of 7.6 cm ± 15.9 cm per patient (Table 1). This corresponds to a mean annual increase of 0.6 ± 1.3 cm/year, or 6 cm per decade. The corresponding median colonic length increased from 191.5 cm to 202.0 cm. The intraperitoneal segments suspended by a mesentery accounted for approximately 80% of this overall mean elongation, 6.1 cm, including an average increase of 2.1 cm in the sigmoid colon and 4.0 cm in the transverse colon. There was no significant net length change for the extraperitoneal segments. Specifically, the rectum increased by 0.3 cm, the descending colon by 0.3 cm, and the ascending colon and cecum by 1.1 cm. A total of 40 (40%) of the 100 patients had a total colonic length increase of at least 10 cm. Figure 2 shows several case examples of the change in colonic length over time from the perspective of the 3D colon map at CTC.

Despite a slightly younger mean age at index CTC for women (53.2 year) compared with men (55.2 years), women had a significantly longer colon at index CTC (202.1 cm vs. 194.0 cm). However, men showed a faster rate of colonic elongation over the observed follow-up interval compared with women (0.88 cm/year vs. 0.31 cm/year; Table 1). These findings resulted in a similar mean total colonic length between men and women at the final CTC examination of 205.4 cm and 206.1 cm, respectively.

**Table 1:** Changes in total colonic length at longitudinal CTC

Cohort	Cases (*n*)	Age at initial CTC (years)	Follow-up interval (years)	Initial colonic length (cm)	Change in colonic length (cm)	Annual change in length (cm)
Total	100	54.2	12.8	198.1	7.6	0.59
Men	50	55.2	12.8	194.0	11.4	0.88
Women	50	53.2	12.8	202.1	4.0	0.31

Mean total colorectal volume was 2261 ml on the initial index CTC examinations and 2285 ml on the final CTC examinations. The corresponding mean value of luminal volume per length was 11.4 ml/cm and 11.1 ml/cm on the initial and final CTC examinations, respectively, which further supports that the overall elongation on the final studies represents a true increase and was not simply related to under-distention on the initial CTC.

## Discussion

Our study provides the most direct evidence to date that colonic length gradually increases in middle-age and older adults. On average, we observed an overall 7.6 cm per decade increase in colonic length, albeit with differences between women and men. Previous and cross-sectional barium enema and CTC studies without longitudinal follow-up had indirectly shown a general trend for longer colons in older adults[6, 12]. Our findings also suggest that the more mobile, intraperitoneal segments (i.e., the transverse and sigmoid colon) account for the majority of this elongation. This seems plausible since the retroperitoneal and extraperitoneal segments are more fixed and presumably less able to lengthen over time. The fact that the cecum has variable mobility and peritoneal extension may also explain why the cecal/ascending segment demonstrated intermediate average lengthening (1.1 cm) between that of the descending colon and rectum (0.3 cm for both) and the transverse and sigmoid colon (4.0 cm and 2.1 cm, respectively). In addition, a previous cadaveric study [1] demonstrated that the traditionally “fixed” retroperitoneal colonic segments often had variable mobility, which could provide more nuanced results.

Previous cross-sectional studies have also noted that females tend to have longer colons [6, 12]. In our study, women had significantly longer colons at the initial index CTC compared with men (202.1 cm vs. 194.0 cm), despite a slightly younger average age (53.2 years vs. 55.2 years). Interestingly, we also found that men demonstrated a faster rate of colonic elongation over the follow-up interval (0.88 cm/year vs. 0.31 cm/year), which essentially closed the gender gap at the final CTC examination (205.4 cm vs. 206.1 cm). Longer colons have also been previously associated with thinner patients[6] and constipation[9]. As such, the relative contributions of sex versus diet are difficult to tease out and warrant further investigation. Constipation is a highly prevalent condition that can adversely affect quality of life[13–15]. As such, the relationship between colonic redundancy and constipation also merit further consideration. Regardless, these colon length findings may also have relevance for optical colonoscopy. For example, an increased rate of incomplete colonoscopy has been noted in thinner women [16]. Not surprisingly, longer colons in general are also associated prior a history of incomplete colonoscopy, as measured by CTC [7].

Our work demonstrates the advantages of using CTC to accurately measure colon length, which complement other relative advantages of CTC for colorectal cancer screening that have been previously enumerated [17–20]. Specifically, CTC allows for precise assessment of the prepared and distended colon in three-dimensional space, easily accounting for the challenging anatomy with an automated software tool that provide a luminal centerline. Traditional methods for measuring colonic length are much more challenging and imprecise. Cadaveric assessment is limited by the obvious physical access issues and post-mortem rigidity[1]. Laparotomy suffers from similar access issues, which are compounded by adhesions and extraperitoneal segments [3]. Contrast enema examinations provide only planar two-dimensional estimation of a complex 3D structure [2, 12]. Finally, colonoscopy is limited by the effect of pleating and telescoping of the bowel, which artificially shortens the colon [5]. Given these limitations, all of these other modalities are generally inaccurate and tend to underestimate the true colonic length. Typical mean colonic lengths measured at optical colonoscopy, laparoscopy, and autopsy are generally foreshortened in the range of 110–130 cm [1, 3–5], whereas at CTC-based mean colonic length in along a 3D luminal centerline is approximately 170 cm, ranging from 150 to 190 cm dep upon the specific population [4–7, 9].

We acknowledge limitations to our investigation. This is a single-center experience involving a predominately White Midwestern U.S. population. We also do not have data on patient bowel habits, such as frequency of bowel movements. We can therefore not specifically comment on the impact of race, ethnicity, geography, or constipation upon colorectal length based on our data. We also did not consider patient BMI, weight change, or history of abdominal surgery. Beyond CTC itself, there is no reliable reference standard to measure colorectal length with which to compare our results. Differences in colonic distention could conceivably impact length measurements. However, because the degree of luminal distention per unit length was slightly less on the final CTC examination compared with the initial one, if anything the degree of elongation over time may be slightly underestimated. Finally, in retrospect it might have been interesting to separate the length measurements for the ascending colon and cecum to see if the variable mobility of the latter accounted for the slightly increased elongation relative to the descending colon and rectum.

In summary, serial in vivo colonic examination with CTC demonstrates a measurable increase in colonic length in middle-age and older adults of approximately 7–8 cm per decade on average. The more mobile intraperitoneal colonic segments (transverse > sigmoid) appear to account for most of this elongation and relevant differences between the sexes were observed.

## Data Availability

All data supporting the findings of this study are available within the paper and its Supplementary Information.
